# Combined application of plant growth-promoting bacteria and iron oxide nanoparticles ameliorates the toxic effects of arsenic in Ajwain (*Trachyspermum ammi* L.)

**DOI:** 10.3389/fpls.2022.1098755

**Published:** 2022-12-28

**Authors:** Yan Sun, Li Ma, Jing Ma, Bingkun Li, Yanfeng Zhu, Fu Chen

**Affiliations:** ^1^ School of Public Administration, Hohai University, Nanjing, China; ^2^ School of Environmental Science and Spatial Informatics, China University of Mining and Technology, Xuzhou, China

**Keywords:** herbaceous crop, heavy metal, *Providencia vermicola*, nano-technology, electron microanalysis

## Abstract

Soil contamination with toxic heavy metals [such as arsenic (As)] is becoming a serious global problem because of the rapid development of the social economy. Although plant growth-promoting bacteria (PGPB) and nanoparticles (NPs) are the major protectants to alleviate metal toxicity, the study of these chemicals in combination to ameliorate the toxic effects of As is limited. Therefore, the present study was conducted to investigate the combined effects of different levels of *Providencia vermicola* (5 ppm and 10 ppm) and iron oxide nanoparticles (FeO-NPs) (50 mg/l^–1^ and 100 mg/l^–1^) on plant growth and biomass, photosynthetic pigments, gas exchange attributes, oxidative stress and response of antioxidant compounds (enzymatic and non-enzymatic), and their specific gene expression, sugars, nutritional status of the plant, organic acid exudation pattern As accumulation from the different parts of the plants, and electron microscopy under the soil, which was spiked with different levels of As [0 μM (i.e., no As), 50 μM, and 100 μM] in Ajwain (*Trachyspermum ammi* L.) seedlings. Results from the present study showed that the increasing levels of As in the soil significantly (*p*< 0.05) decreased plant growth and biomass, photosynthetic pigments, gas exchange attributes, sugars, and nutritional contents from the roots and shoots of the plants, and destroyed the ultra-structure of membrane-bound organelles. In contrast, increasing levels of As in the soil significantly (*p*< 0.05) increased oxidative stress indicators in term of malondialdehyde, hydrogen peroxide, and electrolyte leakage, and also increased organic acid exudation patter in the roots of *T*. *ammi* seedlings. The negative impact of As toxicity can overcome the application of PGPB (*P. vermicola*) and FeO-NPs, which ultimately increased plant growth and biomass by capturing the reactive oxygen species, and decreased oxidative stress in *T*. *ammi* seedlings by decreasing the As contents in the roots and shoots of the plants. Our results also showed that the FeO-NPs were more sever and showed better results when we compared with PGPB (*P. vermicola*) under the same treatment of As in the soil. Research findings, therefore, suggest that the combined application of *P. vermicola* and FeO-NPs can ameliorate As toxicity in *T*. *ammi* seedlings, resulting in improved plant growth and composition under metal stress, as depicted by balanced exudation of organic acids.

## 1 Introduction

Metal contamination issues are becoming increasingly common in China and elsewhere, with many documented cases of metal toxicity in mining industries, foundries, smelters, coal-burning power plants, and agriculture (Afzal et al., 2020; Saleem et al., 2020a; Akram et al., 2022; Al Jabri et al., 2022; El-Kazzaz et al., 2022; Kareem et al., 2022). Heavy metal accumulation in soils is of great concern in agricultural production because of its adverse effects on food safety and marketability, crop growth due to phytotoxicity, and the environmental health of soil organisms (Rehman et al., 2020; Zaheer et al., 2020a; Zaheer et al., 2020b; Ashraf et al., 2022; Chen et al., 2022; Yasmeen et al., 2022; Ma et al., 2022a; Ma et al., 2022b; Ma et al., 2022c; Ma et al., 2022d; Ma et al., 2022e). Arsenic (As) is a highly toxic and carcinogenic element (Irshad et al., 2021; Alsafran et al., 2022b), and the most widespread sources of As in soil and water are natural sources, such as volcanic activities, weathering, erosion of minerals and rocks, and geothermal waters (Bhat et al., 2022; Ulhassan et al., 2022a; Ulhassan et al., 2022b). In addition, use of pesticides, fertilizers, industrial wastes, agricultural chemicals, and copper chromate-arsenate wood preservative are the major anthropogenic sources of As contamination in soil and water (Mondal et al., 2021; Saleem et al., 2022; Tanveer et al., 2022). There is an abundance of evidence that As negatively interferes with several biochemical and physiological processes within a plant, causing reduced plant growth and yield (Farooq et al., 2015; Shahid et al., 2019). Inside the plant cell, heavy metals induce oxidative stress by enhanced production of reactive oxygen species (ROS), which may cause cell death *via* oxidative processes, such as protein oxidation, enzyme inhibition, DNA and RNA damage, and lipid peroxidation (Mumtaz et al., 2021; Tariq et al., 2021; Ali et al., 2022d). Antioxidants, such as superoxide dismutase (SOD), peroxidase (POD), catalase (CAT), and ascorbate peroxidase (APX), come into play to scavenge ROS. For example, SOD facilitates the conversion of superoxide (O^−1^) radicals to hydrogen peroxide (H_2_O_2_), whereas POD decomposes H_2_O_2_ into water (H_2_O) and molecular O_2_ (Afzal et al., 2020; Saleem et al., 2020c; Saleem et al., 2020e; Salam et al., 2022; Ali et al., 2022b). The sites contaminated with As need immediate attention because of the associated severe health risks.

Recently, nanotechnology has gained significant attention because of its widespread application in numerous industries (Hamzah Saleem et al., 2022; Dola et al., 2022). Nanofertilizers could be a favorable methodology, as chemical fertilizers are utilized in very small amounts by plants and other fertilizers left over in the soil can cause environmental risks (Ahmad et al., 2022; Al-Zaban et al., 2022; Ali et al., 2022a; Ali et al., 2022b). The use of nanoparticles (NPs) as a fertilizer may be effective because of decreased nutrient losses in fertilization (Siddiqui et al., 2020). Recently, interest is diverting toward the use of metal-based NPs [e.g., iron oxide nanoparticles (FeO-NPs)] in the agriculture sector (Tanveer et al., 2022). In recent years, several studies on the effects of NPs on plants under abiotic stresses have been accomplished (Rizwan et al., 2019; Ahmed Rather et al., 2021; Ali et al., 2022a). These reports showed that the response of NPs on metal uptake varies with the type of NPs, plants species, and metal species. Plant growth-promoting bacteria (PGPB) help in improving the plant growth and metal resistance (Ali et al., 2022a) by modifying the concentration of growth regulators and phytohormones that facilitate the plant’s ability to tolerate metal contaminants (Rizwan et al.;Saleem et al., 2020d; Ali et al., 2022b). PGPB improve As bioremediation because of their ability to enhance heavy metal bioavailability, uptake, and conversion into less toxic forms through methylation, oxidation, demethylation, and reduction (Panhwar et al., 2020; Ali et al., 2021; Manghwar et al., 2021; Ali et al., 2022b).

Ajwain (*Trachyspermum ammi* L.), a member of the Apiaceae family, is a herbaceous crop plant and widely cultivated in Pakistan, India, Egypt, Iran, and many European countries (Jalbani et al., 2016; Javed et al., 2020). Seeds of *T. ammi* contain beneficial essential oil, traditionally used for different ailments and applications, such as antiseptic, diuretic, antimicrobial, antiviral, bronchodilatory, and hepatoprotective (Rao and Ikram, 2015). *T. amm*i has also been established as an important medicinal plant. During recent years, certain heavy metals have received considerable attention on plant morphology and physiology because of increasing environmental exposure, which also likely to have an negative impact on medicinal plants, including *T. ammi* (Ashraf and Orooj, 2006). Previously, few studies on *T. ammi* were executed to investigate its morphology and physiology under metal stress (Ahad et al., 2014; Rao and Ikram, 2015); but synergistic application of NPs and PGRB on various morpho-physiological characteristics, ionomics, electron microscopy and organic acid exudation potential of *T*. *ammi* was rarely investigated under metal stressed regimes. Therefore, the present study was conducted to study (1) the effect of different levels of PGPB (*Providencia vermicola*) and FeO-NPs on plant growth, biomass, and gaseous exchange parameters of *T. ammi* seedlings under As stress, (2) oxidative stress and the responses of different antioxidative enzymes (enzymatic and non-enzymatic), as well as the response of the specific gene expression; (3) essential minerals uptake, organic acids exudation, and As accumulation in different organs of *T. ammi* seedlings under As stress; and (4) electron microscopy of membrane-bound organelles of *T. ammi* seedlings grown under varying levels of As in the soil.

## 2 Materials and methods

### 2.1 Experimental setup

A pot experiment was conducted at the Botanical Garden of the Hohai University, Nanjing, China. Viable seeds of Ajwain (*T*. *ammi*) were collected from Hohai University. Before sowing, the seeds were carefully washed and sterilized in 0.1% mercuric chloride solution for 1 min and then washed three times with distilled water. The soil sample was air dried, passed through a 5-mm sieve, and was water saturated two times before being used in pots. The soil used for this study was collected from the experimental stations of Hohai University, and its properties was as follow: pH-6.9, EC-0.9 dS cm^−1^, organic matter-17 g kg^−1^, EK-21 mg kg^−1^, TP-0.17 g kg^−1^ and TN-16 g kg^−1^ (further details are mentioned in **Table S1**). All pots were divided in the three subgroups: (1) without any As treatment, (2) addition of 50 µM of As, and (3) addition of 100 µM of As. All of the As-treated pots were further treated with FeO-NPs (50 mg/l^–1^ and 100 mg/l^–^1) and PGPB (*P. vermicola;* 5 ppm and 10 ppm) to evaluate their ameliorative effects on As stress. Thus, all pots were divided into the following 15 treatments: (1) As (0 μM), FeO-NPs (0 mg/l^–1^), *P. vermicola* (0 ppm); (2) As (0 μM), FeO-NPs (50 mg/l^–1^), *P. vermicola* (0 ppm); (3) As (0 μM), FeO-NPs (100 mg/l^–1^), *P. vermicola* (0 ppm); (4) As (0 μM), FeO-NPs (0 mg/l^–1^), *P. vermicola* (5 ppm); (5) As (0 μM), FeO-NPs (0 mg/l^–1^), *P. vermicola* (10 ppm); (6) As (50 μM), FeO-NPs (0 mg/l^–1^), *P. vermicola* (0 ppm); (7) As (50 μM), FeO-NPs (50 mg/l^–1^), *P. vermicola* (0 ppm); (8) As (50 μM), FeO-NPs (100 mg/l^–1^), *P. vermicola* (0 ppm); (9) As (50 μM), FeO-NPs (0 mg/l^–1^), *P. vermicola* (5 ppm); (10) As (50 μM), FeO-NPs (0 mg/l^–1^), *P. vermicola* (10 ppm); (11) As (100 μM), FeO-NPs (0 mg/l^–1^), *P. vermicola* (0 ppm); (12) As (100 μM), FeO-NPs (50 mg/l^–1^), *P. vermicola* (0 ppm); (13) As (100 μM), FeO-NPs (100 mg/l^–1^), *P. vermicola* (0 ppm); (14) As (100 μM), FeO-NPs (0 mg/l^–1^), *P. vermicola* (5 ppm); and (15) As (100 μM), FeO-NPs (0 mg/l^–1^), *P. vermicola* (10 ppm). The salt of sodium arsenate (Na_2_HAsSO_4_.7H_2_O; Sigma-Aldrich, St. Louis, MO, USA) was used to artificially spike natural soil at various levels [i.e., 0 µM (no As), 50 µM and 100 µM]. After adding As, the pots were equilibrated for 2 months, with four cycles of saturation with distilled water and air drying. Application of FeO-NPs and *P. vermicola* was provided soon after the seed germination (2 weeks after the seed sowing). The concentration of *P. vermicola* was screened under the As stress and best levels were chosen by following a previous study (Tanveer et al., 2022). The levels of FeO-NPs (50 mg/l^–1^ and 100 mg/l^–1^) were selected by following the application levels of a recent report (Manzoor et al., 2021). In addition, (Tanveer et al., 2022) 14 different strains of PGPB were studied, and *P. vermicola* was selected on the basis of 16S rRNA gene sequencing and it showing maximum resistance against As stress. The extracellular biosynthesis of FeO-NPs using the RNT4 strain was carried out according to Fatemi et al. (Fatemi et al., 2018). Specifically, the RNT4 strain was cultivated with a broth (NB) media in a shaker incubator at 28 ± 2°C and 170 rpm for 24 h. Afterwards, the supernatant was obtained after centrifugation at 6000 *g* for 10 min. For the biosynthesis of FeO-NPs, 50 ml of the supernatant of RNT4 culture was added into an equal volume of 5-mM ferric chloride hexahydrate (FeCl_3_.6H2O) solution in a 250-ml Erlenmeyer flask. The reaction mixture was incubated under dark condition in a shaking incubator at 28 ± 2°C and 170 rpm for 24 h. The yielded FeO-NPs were collected using centrifugation at 10,000 g for 15 min after a color change from pale yellow to turbid brown. Irrigation with As-free water and other intercultural operations were performed when needed. All pots were placed in completely randomized design, having five plants in each pot with four replicates of each treatment. The total duration of experimental treatments was 4 weeks under controlled conditions. All plants in the glass house territory received natural light, with a day/night temperature of 35/40°C and a day/night humidity of 60/70%. All chemicals used were of analytical grade, procured from Sinopharm Chemical Reagent Co., Ltd (Shanghai, China).

### 2.2 Analysis of samples and data collection

After 4 weeks, the remaining three seedlings were uprooted and washed gently with the help of distilled water to eliminate the aerial dust and deposition. Hohai University laboratories were employed for the determination of soil parameters. Functional leaf in each treatment was picked at a rapid growth stage during 09:00–10:30. The sampled leaves were washed with distilled water, immediately placed in liquid nitrogen, and stored in a freezer at –80°C for further analysis. All of the harvested plants were divided into two parts (i.e., roots and shoots) to study different physio-biochemical traits. Leaves from each treatment group were picked for chlorophyll, carotenoid, oxidative stress, and antioxidants analysis. Root and shoot lengths were measured straightway after the harvesting by using measuring scale and digital weighting balance to measure fresh biomass. Roots were uprooted and immersed in 20-mM ethylenediaminetetraacetic acid disodium salt (Na_2_EDTA) for 15–20 min to remove As adhered to the root surfaces. Then, roots were washed thrice with distilled water and, finally, once with de-ionized water, and dried for further analysis. The different parts of the plant (i.e., roots and shoots) were oven-dehydrated at 65°C for 72 h for As determination, and the total plant dry weight was also measured. Although this experiment was conducted in pots, for the collection of organic acids, two seedlings were transferred to the rhizoboxes, which consist of a plastic sheet, a nylon net and wet soil (properties of soil are given in **Table S1**) (Javed et al., 2013). After 48 h, plants were taken from the rhizoboxes and the roots were washed with redistilled water to collect the exudates from root surface. The samples were filtered through a 0.45-μm filter (Millex-HA, Millipore) and collected in eppendorf tubes (Greger and Landberg, 2008). The collected samples were mixed with sodium hydroxide (NaOH) (0.01 M) to analyze the organic acids. However, the samples used for analysis of oxalic acid were not treated with NaOH.

### 2.3 Determination of photosynthetic pigments and gas exchange characteristics

Leaves were collected for the determination of chlorophyll and carotenoid contents. For chlorophylls, 0.1 g of fresh leaf sample was extracted with 8 ml of 95% acetone for 24 h at 4°C in the dark. The absorbance was measured by a spectrophotometer (UV-2550; Shimadzu, Kyoto, Japan) at 646.6 nm, 663.6 nm, and 450 nm. Chlorophyll content was calculated by the standard method (Mehmood et al., 2021).

Net photosynthesis (*Pn*), leaf stomatal conductance *(Gs)*, transpiration rate (*Ts*), and intercellular carbon dioxide concentration (*Ci*) were measured from four different plants in each treatment group. Measurements were conducted between 11:30 and 13:30 on days with a clear sky. Rates of leaf *Pn*, *Gs*, *Ts*, and *Ci* were measured with a LI-COR gas-exchange system (LI-6400; LI-COR Biosciences, Lincoln, NE, USA) with a red-blue LED light source on the leaf chamber. In the LI-COR cuvette, carbon dioxide (CO_2_) concentration was set as 380 mmol/mol^–1^ and LED light intensity was set at 1000 mmol m^–2^ s^–1^, which was the average saturation intensity for photosynthesis in *Spinacia oleracea* (Austin, 1990).

### 2.4 Determination of oxidative stress indicators

The degree of lipid peroxidation was evaluated as malondialdehyde (MDA) contents. Briefly, 0.1 g of frozen leaves were ground at 4°C in a mortar with 25 ml of 50-mM phosphate buffer solution (pH 7.8) containing 1% polyethene pyrrole. The homogenate was centrifuged at 10,000 × g at 4°C for 15 min. The mixtures were heated at 100°C for 15–30 min and then quickly cooled in an ice bath. The absorbance of the supernatant was recorded by using a spectrophotometer (xMark™ Microplate Absorbance Spectrophotometer; Bio-Rad, Hercules, CA, USA) at wavelengths of 532 nm, 600 nm, and 450 nm. Lipid peroxidation was expressed as l mol g^−1^ by using the formula: 6.45 (A532 – A600) – 0.56 A450. Lipid peroxidation was measured by using a method previously published by Heath and Parker (Heath and Packer, 1968).

To estimate the H_2_O_2_ content of plant tissues (i.e., root and leaf), 3 ml of sample extract was mixed with 1 ml of 0.1% titanium sulfate in 20% (v/v) H_2_SO_4_ and centrifuged at 6000 × *g* for 15 min. The yellow color intensity was evaluated at 410 nm. The H_2_O_2_ level was computed by the extinction coefficient of 0.28 mmol^−1^ cm^−1^. The contents of H_2_O_2_ were measured by the method presented by Jana and Choudhuri (Jana and Choudhuri, 1981).

Stress-induced electrolyte leakage (EL) of the uppermost stretched leaves was determined by using the methodology of Dionisio-Sese and Tobita (Dionisio-Sese and Tobita, 1998). The leaves were cut into minor slices (5 mm in length) and placed in test tubes of 8 ml of distilled water. The tubes were incubated and transferred into a water bath for 2 h prior to measuring the initial electrical conductivity (EC_1_). The samples were autoclaved at 121°C for 20 min and then cooled down to 25°C before measuring the final electrical conductivity (EC_2_). EL was calculated by the following formula:


$$EL=(EC_{1}/EC_{2})\times 100$$

### 2.5 Determination of antioxidant enzyme activities and relative gene expression

To evaluate enzyme activities, fresh leaves (0.5 g) were homogenized in liquid nitrogen and 5 ml of 50-mmol sodium phosphate buffer (SPB) (pH 7.0), including 0.5-mmol ethylenediaminetetraacetic acid (ETDA) and 0.15-mol sodium chloride. The homogenate was centrifuged at 12,000 × *g* for 10 min at 4°C, and the supernatant was used for measurement of SOD and POD activities. SOD activity was assayed in 3-ml reaction mixture, containing 50-mM SPB (pH 7), 56-mM nitro blue tetrazolium, 1.17-mM riboflavin, 10-mM methionine, and 100-μl enzyme extract. Finally, the sample was measured by using a spectrophotometer (xMark™ Microplate Absorbance Spectrophotometer; Bio-Rad). Enzyme activity was measured by using a method by Chan and Pan, (Chen and Pan, 1996) and expressed as U g^−1^ FW.

POD activity in the leaves was estimated by using the method of Sakharov and Ardila (Sakharov and Ardila, 1999) and by using guaiacol as the substrate. A reaction mixture (3 ml) containing 0.05 ml of enzyme extract, 2.75 ml of 50-mM phosphate buffer (pH 7.0), 0.1 ml of 1% H_2_O_2_, and 0.1 ml of 4% guaiacol solution was prepared. Increases in the absorbance at 470 nm because of guaiacol oxidation was recorded for 2 min. One unit of enzyme activity was defined as the amount of the enzyme.

CAT activity was analyzed according to Aebi (Zainab et al., 2021). The assay mixture (3.0 ml) comprised 100 μl of enzyme extract, 100 μl of H_2_O_2_ (300 mM), and 2.8 ml of 50-mM phosphate buffer with 2 mM ETDA (pH 7.0). CAT activity was measured from the decline in absorbance at 240 nm as a result of H_2_O_2_ loss (*ϵ* = 39.4 mM^−1^ cm^−1^).

APX activity was measured according to Nakano and Asada (Nakano and Asada, 1981). The mixture containing 100 μl of enzyme extract, 100 μl of ascorbate (7.5 mM), 100 μl of H_2_O_2_ (300 mM), and 2.7 ml of 25-mM potassium phosphate buffer with 2-mM EDTA (pH 7.0) was used for measuring APX activity. The oxidation pattern of ascorbate was estimated from the variations in wavelength at 290 nm (*ϵ* = 2.8 mM^−1^ cm^−1^).

Quantitative real-time PCR (RT-qPCR) assay was applied to investigate the expression levels of four stress-related genes [i.e., iron superoxidase dismutase (Fe-SOD), POD, CAT, and APX]. Total RNA was extracted from leaf tissue samples using RNeasy Plant Mini kits (Qiagen, Manchester, UK). Contaminating DNA was then removed and first-strand copy DNAs were prepared using reverse transcription kits (Qiagen). RT-qPCR analysis was conducted in accordance with the protocol of the QuantiTect SYBR Green PCR kit (Qiagen). Reaction volume and PCR amplification conditions were adjusted as mentioned by El-Esawi et al. (El-Esawi et al., 2020). The gene amplifications of Sirhindi et al. (Sirhindi et al., 2016) are given in **Table S2**.

### 2.6 Determination of non-enzymatic antioxidants, sugars, and proline contents

Plant ethanol extracts were prepared for the determination of non-enzymatic antioxidants and some key osmolytes. For this purpose, 50 mg of dry plant material was homogenized with 10 mL of ethanol (80%) and filtered through Whatman No. 41 filter paper. The residue was re-extracted with ethanol, and the two extracts were pooled together to a final volume of 20 ml. The determination of flavonoids (Pękal and Pyrzynska, 2014), phenolics (Ali et al., 2022c), ascorbic acid (Azuma et al., 1999), anthocyanin (Lewis et al., 1998), and total sugars (Dubois et al., 1956) was performed from the extracts.

Fresh leaf material (0.1 g) was mixed thoroughly in 5 ml of aqueous sulphosalicylic acid (3%). The mixture was centrifuged at 10000 × *g* for 15 min, and an aliquot (1 ml) was poured into a test tube having 1 ml of acidic ninhydrin and 1 ml of glacial acetic acid. The reaction mixture was first heated at 100°C for 10 min and then cooled in an ice bath. The reaction mixture was extracted with 4 ml of toluene, and test tubes were vortexed for 20 s and cooled. Thereafter, the light absorbance at 520 nm was measured by using a UV-VIS spectrophotometer (Hitachi U-2910, Tokyo, Japan). The free proline content was determined on the basis of the standard curve at 520 nm absorbance and expressed as µmol (g FW)^−1^ (Bates et al., 1973).

### 2.7 Determination of nutrient content

For nutrient analysis, plant roots and shoots were washed twice in redistilled water, dipped in 20 mM of EDTA for 3 s, and then, again, washed with deionized water twice for the removal of adsorbed metal on the plant surface. The washed samples were then oven-dried for 24 h at 105°C. The dried roots and shoots were digested by using a wet digestion method in nitric acid (HNO_3_) : perchloric acid (HclO_4_) (7 : 3 V/V) until clear samples were obtained. Each sample was filtered and diluted with redistilled water up to 50 ml. The root and shoot contents of iron, calcium, magnesium, and phosphorus were analyzed by using atomic absorption spectrophotometer (AAS) model Agilent 240FS-AA.

### 2.8 Determination of root exudates analysis and arsenic concentration

To determine the concentration of organic acids, freeze-dried exudates were mixed with ethanol (80%), and 20 μl of the solutions were injected into the C18 column (Brownlee Analytical C-183 µm; length 150 mm × 4.6 mm^2^, USA). Quantitative analysis of organic acids in root exudates was executed with high-performance liquid chromatography (HPLC), having a Flexer FX-10 UHPLC isocratic pump (PerkinElmer, Waltham, MA, USA). The mobile phase used in HPLC comprised an acidic solution of aceto-nitrile, containing aceto-nitrile : H_2_SO_4_ : acetic acid in ratios of 15 : 4 : 1, respectively, and pH of 4.9. The samples were analyzed at a flow rate of 1.0 ml min^−1^ for a time period of 10 min. The inner temperature of the column was fixed at 45°C, and quantification of organic acids was carried out at 214 nm wavelength with the help of a detector (UV-VIS Series 200, USA), as described by UdDin et al. (Uddin et al., 2015). Freeze-dried samples were dissolved in redistilled water, and the pH of the exudates was recorded with LL micro-pH glass electrode by using a pH meter (ISTEK Model 4005–08007, Seoul, South Korea).

For the determination of total As concentration in shoots and roots, samples were oven-dried at 65°C for 24 h and ashed in a muffle furnace at 550°C for 20 h. Then, the ash was incubated with 31% (m/v) HNO_3_ and 17.5% (v/v) H_2_O_2_ at 70°C for about 2 h, and added to distilled water. The As concentration in the digest was determined using an AAS.

### 2.9 Transmission electron microscopy

For transmission electron microscopy (TEM), leaf samples were collected and placed in liquid nitrogen. Small sections of the leaves (1–3 mm in length) were fixed in 4% glutaraldehyde (v/v) in 0.2 mol/l of SPB (pH 7.2) for 6–8 h, post-fixed in 1% osmium tetroxide for 1 h, and then in 0.2-mol/l SPB (pH 7.2) for 1–2 h. Samples were dehydrated in a graded ethanol series (50%, 60%, 70%, 80%, 90%, 95%, and 100%) followed by acetone, filtered, and embedded in Spurr resin. Ultra-thin sections (80 nm) were prepared and mounted on copper grids for observation under a transmission electron microscope (JEOL TEM-1200EX) at an accelerating voltage of 60.0 kV or 80.0 kV.

### 2.10 Statistical analysis

The normality of data was analyzed using IBM SPSS software (version 21.0. IBM Corporation, Armonk, NY, USA) through a multivariate *post-hoc* test, followed by a Duncan’s test to determine the interaction among significant values. Thus, the differences between treatments were determined by using ANOVA, the least significant difference test (*p*< 0.05) was used for multiple comparisons between treatment means and, where significant, Tukey’s honestly significant difference *post-hoc* test was used to compare the multiple comparisons of means. The analysis showed that the data in this study were almost normally distributed. The graphical presentation was carried out using Origin-Pro 2017 (OriginLab Corporation, Northampton, UK).

## 3 Results

### 3.1 Ameliorative effects of iron oxide nanoparticles and *Providencia vermicola* on growth and photosynthetic efficiency under arsenic stress

**Figure 1:**
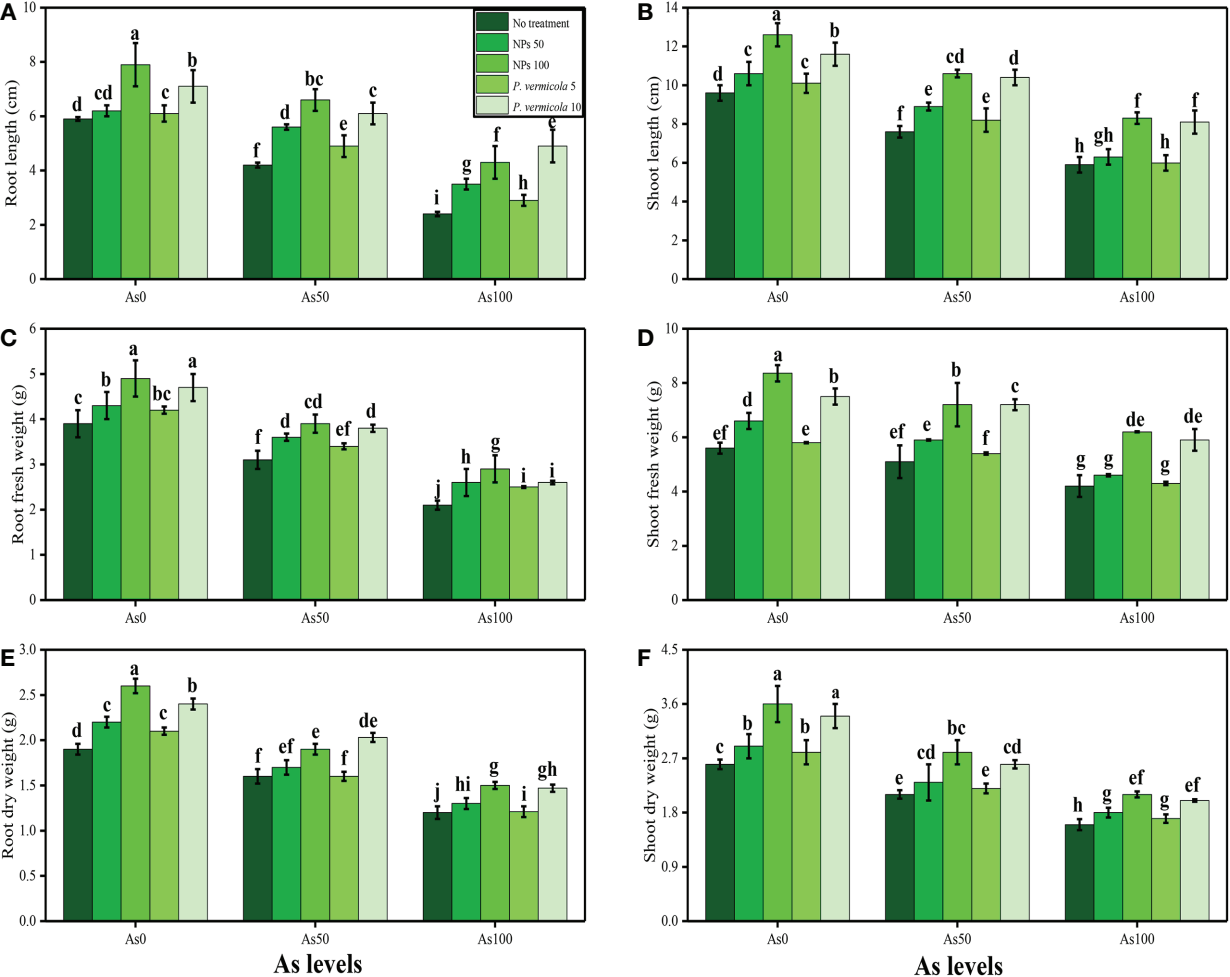
Effect of combined application of various levels of iron oxide nanoparticles (FeO-NPs) (i.e., 50 mg/l^−1^ and 100 mg/l^−1^) and plant growth-promoting bacteria (*Providencia vermicola*) (i.e., 5 ppm and 10 ppm) on root length **(A)**, shoot length **(B)**, root fresh weight **(C)**, shoot fresh weight **(D)**, root dry weight **(E)**, and shoot dry weight **(F)** of Ajwain (*Trachyspermum ammi* seedlings) grown under various stress levels of arsenic (i.e., 0 μM, 50 μM, and 100 μM). Values are demonstrated as means of four replicates, along with standard deviation (SD; *n* = 4). Two-way ANOVA was performed and means differences were tested by Tukey’s highly significant difference *post-hoc* test (*p*< 0.05). Different lowercase letters on the error bars indicate significant difference between the treatments.

**Figure 2:**
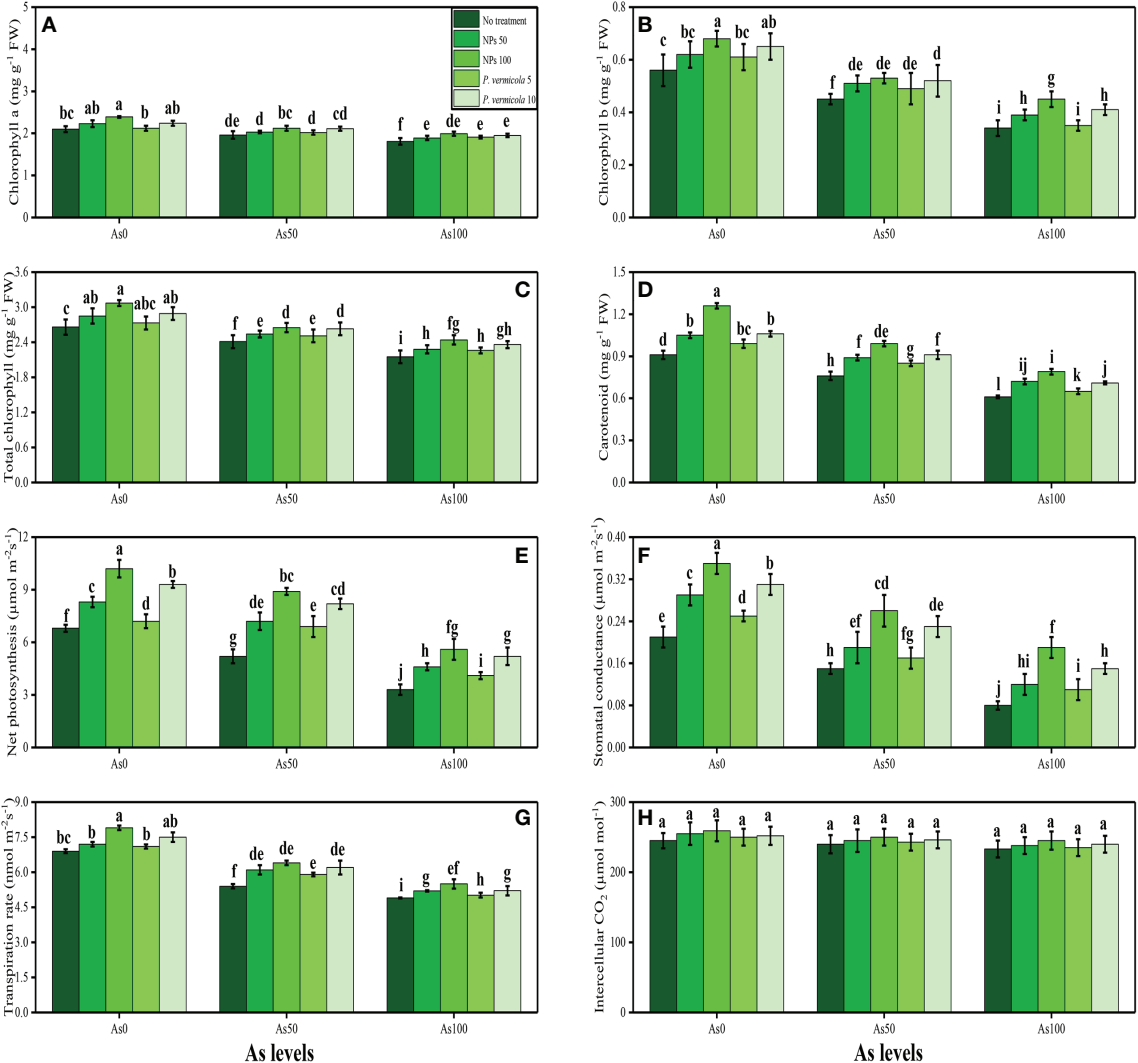
Effect of combined application of various levels of iron oxide nanoparticles (FeO-NPs) (i.e., 50 mg/l^−1^ and 100 mg/l^−1^) and plant growth-promoting bacteria (*Providencia vermicola*) (i.e., 5 ppm and 10 ppm) on chlorophyll-a content **(A)**, chlorophyll-b content **(B)**, total chlorophyll content **(C)**, carotenoid content **(D)**, net photosynthesis, **(E)** stomatal conductance **(F)**, transpiration rate **(G)**, and intercellular CO_2_ **(H)** of Ajwain (*Trachyspermum ammi* seedlings) grown under various stress levels of arsenic (i.e., 0 μM, 50 μM, and 100 μM). Values are demonstrated as means of four replicates along with standard deviation (SD; *n* = 4). Two-way ANOVA was performed and means differences were tested by Tukey’s highly significant difference *post-hoc* test (*p*< 0.05). Different lowercase letters on the error bars indicate significant difference between the treatments.

In the present study, various growth and photosynthetic parameters were also measured in *T*. *ammi* seedlings grown under the different levels of As [i.e., 0 µM (no As), 50 µM, and 100 µM] in the soil, which was also supplemented with the different levels of *P. vermicola* (i.e., 5 ppm and 10 ppm) and FeO-NPs (i.e., 50 mg/l^−1^ and 100 mg/l^−1^). The data regarding shoot length, root length, shoot fresh weight, root fresh weight, shoot dry weight, and root dry weight are presented in **Figure 1**, and the data regarding the content of chlorophyll-a, chlorophyll-b, total chlorophyll, carotenoid content, net photosynthesis, stomatal conductance, transpiration rate, and intercellular CO_2_ are presented in **Figure 2**. According to the results, it was noticed that the increasing levels of As in the soil significantly (*p*< 0.05) decreased plant growth and biomass and photosynthetic pigments in *T*. *ammi* seedlings without the application of *P*. *vermicola* and FeO-NPs (**Figures 1**, **2**). According to the given results, increasing levels of As (i.e., 50 µM and 100 µM) in the soil significantly (*p*< 0.05) decreased shoot length, root length, shoot fresh weight, root fresh weight, shoot dry weight, root dry weight, chlorophyll-a, chlorophyll-b, total chlorophyll, carotenoid content, net photosynthesis, stomatal conductance, and transpiration rate in *T*. *ammi* seedlings, compared with the plants grown without the treatment of As in the soil. The exogenous application of *P*. *vermicola* and FeO-NPs was also performed to measure various growth (**Figure 1**) and photosynthetic attributes (**Figure 2**) in *T*. *ammi* seedlings under the elevating levels of As in the soil. The application of *P*. *vermicola* and FeO-NPs non-significantly increased shoot length, root length, shoot fresh weight, root fresh weight, shoot dry weight, root dry weight, chlorophyll-a, chlorophyll-b, total chlorophyll, carotenoid content, net photosynthesis, stomatal conductance, and transpiration rate at all levels of As in the soil, compared with the plants that were grown without the application of *P*. *vermicola* and FeO-NPs. Our results also showed that the FeO-NPs was more severe and showed better results when we compared with PGPB (*P. vermicola*) under the same treatment of As in the soil. We also noticed that As toxicity did not significantly affect the intercellular CO_2_ levels, and that application of *P*. *vermicola* and FeO-NPs did not significantly influence the intercellular CO_2_ levels in *T*. *ammi* seedlings under all levels of As in the soil (**Figure 2H**).

### 3.2 Ameliorative effects of iron oxide nanoparticles and *Providencia vermicola* on oxidative stress and antioxidant capacity under arsenic stress

**Figure 3:**
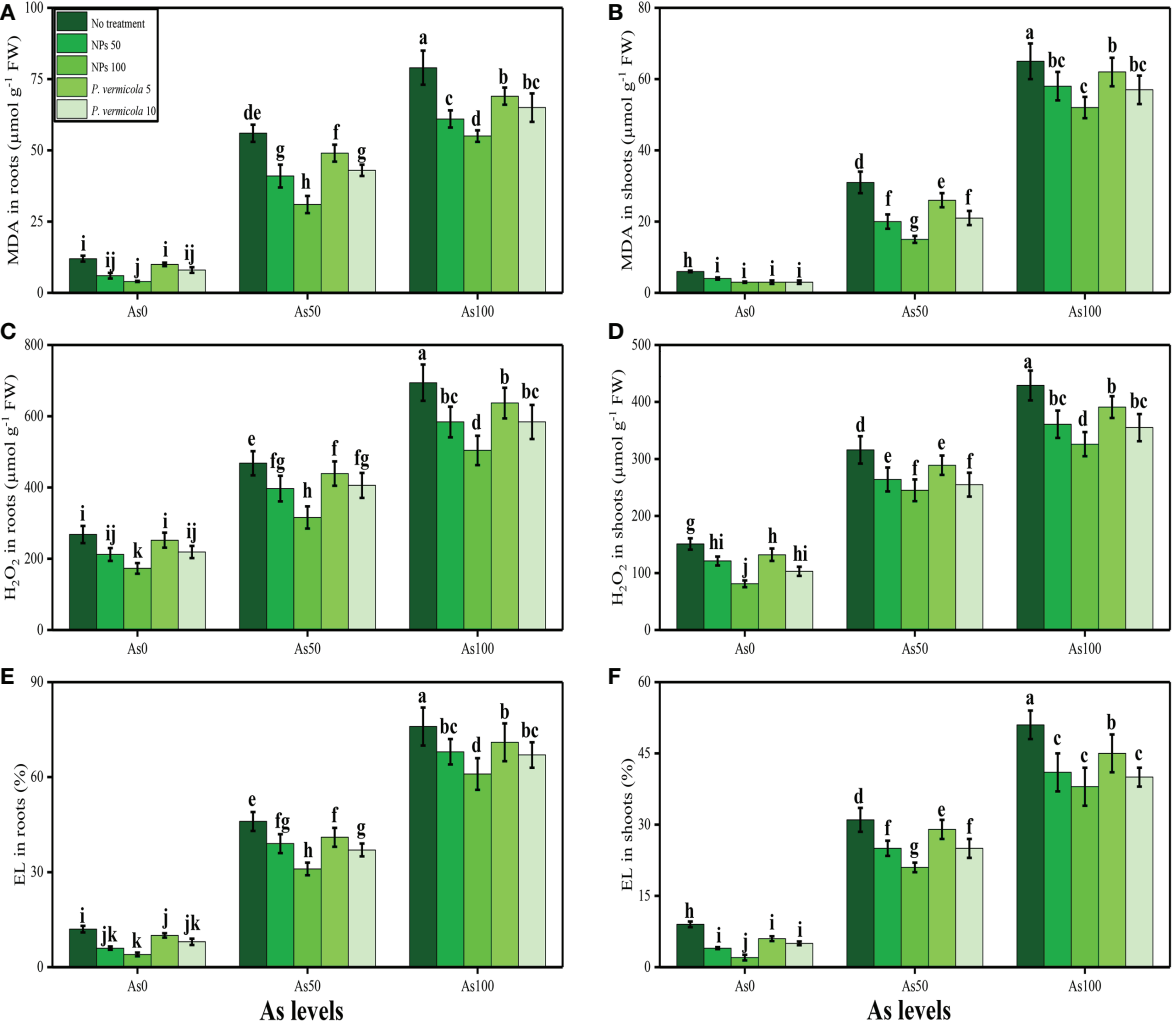
Effect of combined application of various levels of iron oxide nanoparticles (FeO-NPs) (i.e., 50 mg/l^−1^ and 100 mg/l^−1^) and plant growth-promoting bacteria (*Providencia vermicola*) (i.e., 5 ppm and 10 ppm) on malondialdehyde (MDA) contents in the roots **(A)**, MDA contents in the leaves **(B)**, hydrogen peroxide (H_2_O_2_) contents in the roots **(C)**, H_2_O_2_ contents in the leaves **(D)**, EL percentage in the roots **(E)**, and EL percentage in the leaves **(F)** of Ajwain (*Trachyspermum ammi* seedlings) grown under various stress levels of arsenic (i.e., 0 μM, 50 μM, and 100 μM). Values are demonstrated as means of four replicates along with standard deviation (SD; *n* = 4). Two-way ANOVA was performed and means differences were tested by Tukey’s highly significant difference *post-hoc* test (*p*< 0.05). Different lowercase letters on the error bars indicate significant difference between the treatments.

MDA content, H_2_O_2_ initiation, and EL (%) increased in the roots and shoots of *T*. *ammi* seedlings with the increasing concentrations of As (i.e., 50 µM and 100 µM) in the soil medium without *P*. *vermicola* and FeO-NPs when compared with plants grown in 0 µM of As in the soil. The data regarding oxidative stress indicators in roots and shoots of *T*. *ammi* seedlings are presented in **Figure 3**. It was observed that the contents (%) of MDA, H_2_O_2_, and EL were increased in the roots and also in the shoots grown in 100 µM of As without the application of *P*. *vermicola* and FeO-NPs when compared with plants grown in 0 µM of As in the soil without the application of *P*. *vermicola* and FeO-NPs. Application of *P*. *vermicola* and FeO-NPs significantly (*p*< 0.05) decreased the contents (%) of MDA, H_2_O_2_, and EL in the roots and also in the shoots of the plants grown with As level of 100 µM under *P*. *vermicola* and FeO-NPs application when compared with plants grown with 100 µM of chromium (Cr) without the application of *P*. *vermicola* and FeO-NPs.

**Figure 4:**
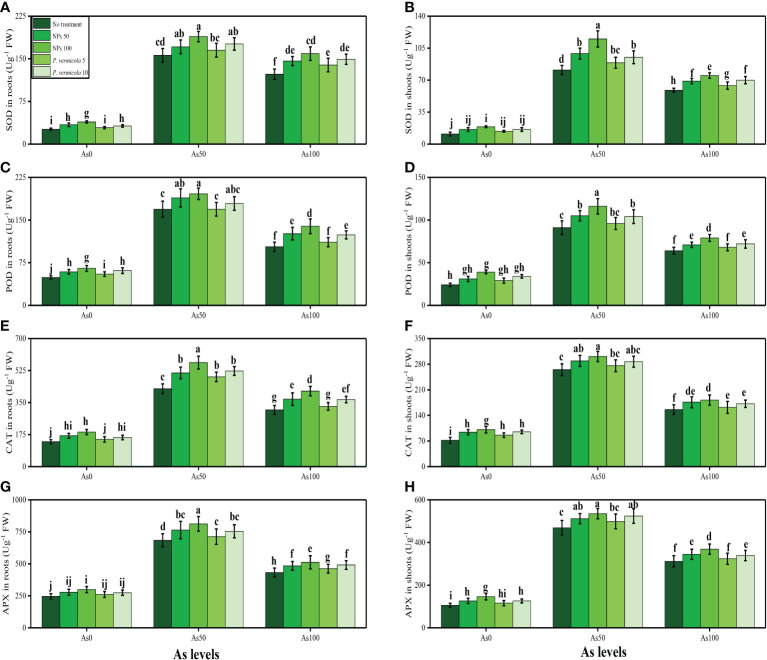
Effect of combined application of various levels of iron oxide nanoparticle (FeO-NPs) (i.e., 50 mg/l^−1^ and 100 mg/l^−1^) and plant growth-promoting bacteria (*Providencia vermicola*) (i.e., 5 ppm and 10 ppm) on superoxide dismutase (SOD) activity in the roots **(A)**, SOD activity in the shoots **(B)**, peroxidase (POD) activity in the roots **(C)**, POD activity in the shoots **(D)** catalase (CAT) activity in the roots **(E)**, CAT activity in the shoots **(F)**, ascorbate peroxidase (APX) activity in the roots, **(G)** and APX activity in the shoots **(H)** of Ajwain (*Trachyspermum ammi* seedlings) grown under various stress levels of arsenic (i.e., 0 μM, 50 μM, and 100 μM). Values are demonstrated as means of four replicates along with standard deviation (SD; *n* = 4). Two-way ANOVA was performed and means differences were tested by Tukey’s highly significant difference *post-hoc* test (*p*< 0.05). Different lowercase letters on the error bars indicate significant difference between the treatments.

**Figure 5:**
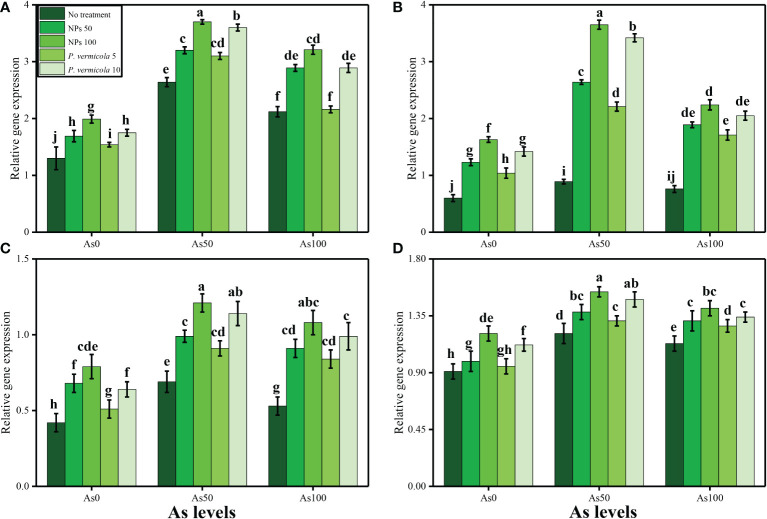
Effect of combined application of various levels of iron oxide nanoparticles (FeO-NPs) (i.e., 50 mg/l^−1^ and 100 mg/l^−1^) and plant growth-promoting bacteria (*Providencia vermicola*) (i.e., 5 ppm and 10 ppm) on iron superoxidase dismutase (Fe-SOD) **(A)**, peroxidase (POD) **(B)**, catalase (CAT) **(C)**, and ascorbate peroxidase (APX) **(D)** of Ajwain (*Trachyspermum ammi* seedlings) grown under various stress levels of arsenic (i.e., 0 μM, 50 μM, and 100 μM). Values are demonstrated as means of four replicates along with standard deviation (SD; *n* = 4). Two-way ANOVA was performed and means differences were tested by Tukey’s highly significant difference *post-hoc* test (*p*< 0.05). Different lowercase letters on the error bars indicate significant difference between the treatments.

**Figure 6:**
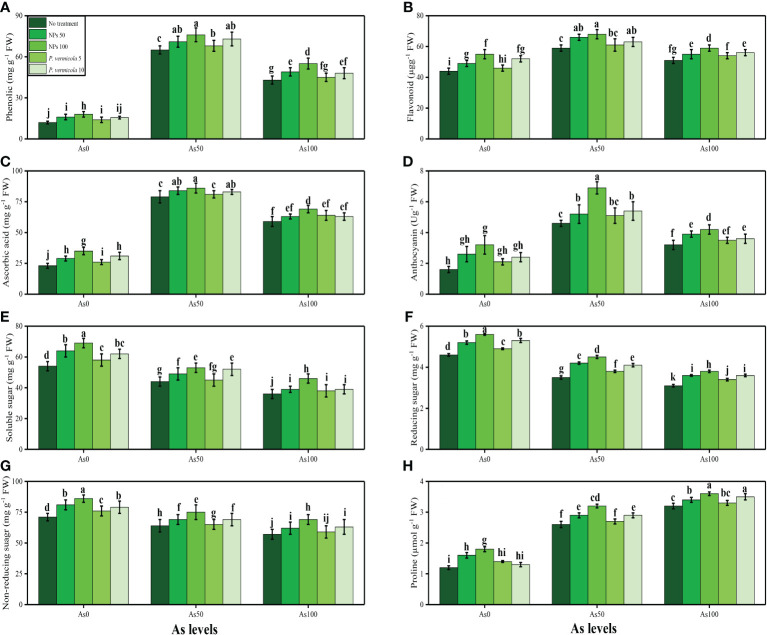
Effect of combined application of various levels of iron oxide nanoparticles (FeO-NPs) (i.e., 50 mg/l^−1^ and 100 mg/l^−1^) and plant growth-promoting bacteria (*Providencia vermicola*) (i.e., 5 ppm and 10 ppm) on phenolic contents **(A)**, flavonoid contents **(B)**, ascorbic acid contents **(C)**, anthocyanin contents **(D)**, soluble sugar contents **(E)**, reducing sugar contents **(F)**, non-reducing sugar contents **(G)**, and proline contents **(H)** of Ajwain (*Trachyspermum ammi* seedlings) grown under various stress levels of arsenic (i.e., 0 μM, 50 μM, and 100 μM). Values are demonstrated as means of four replicates along with standard deviation (SD; *n* = 4). Two-way ANOVA was performed and means differences were tested by Tukey’s highly significant difference *post-hoc* test (*p*< 0.05). Different lowercase letters on the error bars indicate significant difference between the treatments.

The activities of various antioxidant enzymes, such as SOD, POD, CAT, and APX, in the roots and shoots of *T*. *ammi* seedlings and their specific gene expression (i.e., Fe-SOD, POD, CAT, and APX), and the content of non-enzymatic compounds, such as phenolic, flavonoid, ascorbic acid, and anthocyanin, were also measured in the present study. The data regarding the activities of enzymatic antioxidants (SOD, POD, CAT, and APX) are presented in **Figure 4**, and their specific gene expression are presented in **Figure 5**. The results regarding the content of non-enzymatic antioxidants (phenolic, flavonoid, ascorbic acid, and anthocyanin) are presented in **Figure 6**. The results showed that the activities of enzymatic antioxidants (SOD, POD, CAT, and APX) and their specific gene expression and also the contents of non-enzymatic antioxidants (phenolic, flavonoid, ascorbic acid, and anthocyanin) were increased up to an As level of 50 µM in the soil, but decreased gradually after an As level of 100 µM in the soil, compared with the control treatment. The results also showed that the addition of *P*. *vermicola* and FeO-NPs non-significantly increased the activities of enzymatic antioxidants (SOD, POD, CAT, and APX), their specific gene expression, and the content of non-enzymatic antioxidants (phenolic, flavonoid, ascorbic acid, and anthocyanin) at all levels of As [i.e., 0 µM (no As), 50 µM, and 100 µM] in the soil, compared with plants that were grown in the soil not supplemented with *P*. *vermicola* and FeO-NPs.

### 3.3 Ameliorative effects of iron oxide nanoparticles and *Providencia vermicola* on sugar and nutrient uptake under arsenic stress

**Figure 7:**
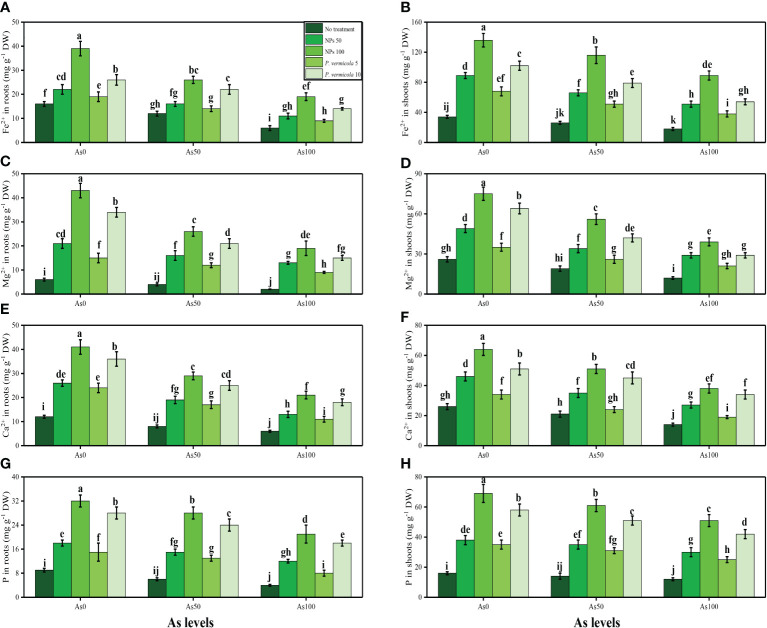
Effect of combined application of various levels of iron oxide nanoparticles (FeO-NPs) (i.e., 50 mg/l^−1^ and 100 mg/l^−1^) and plant growth-promoting bacteria (*Providencia vermicola*) (i.e., 5 ppm and 10 ppm) on iron contents in the roots **(A)**, iron contents in the shoots **(B)**, magnesium contents in the roots **(C)**, magnesium contents in the shoots **(D)**, calcium contents in the roots **(E)**, calcium contents in the shoots **(F)**, phosphorus contents in the roots **(G)**, and phosphorus contents in the shoots **(H)** of Ajwain (*Trachyspermum ammi* seedlings) grown under various stress levels of arsenic (0 i.e., 0 μM, 50 μM, and 100 μM). Values are demonstrated as means of four replicates along with standard deviation (SD; *n* = 4). Two-way ANOVA was performed and means differences were tested by Tukey’s highly significant difference *post-hoc* test (*p*< 0.05). Different lowercase letters on the error bars indicate significant difference between the treatments.

The content of soluble sugar, reducing sugar, non-reducing sugar, proline, and various nutrients, such as calcium (Ca^2+^), magnesium (Mg^2+^), iron (Fe^2+^), and phosphorus (P), were also measured in the roots and shoots of *T*. *ammi* seedlings in the present study under the different levels of As [i.e., 0 µM (no As), 50 µM, and 100 µM] in the soil that was also supplemented with *P*. *vermicola* and FeO-NPs. The data regarding the content of soluble sugar, reducing sugar, non-reducing sugar, and proline are presented in **Figure 6**, and the data regarding the content of Ca^2+^, Mg^2+^, Fe^2+^, and P in the roots and shoots of the plants are presented in **Figure 7**. The results of the present study show that the increasing levels of As in the soil significantly (*p*< 0.05) decreased the content of nutrients (Ca^2+^, Mg^2+^, Fe^2+^, and P) in the roots and shoots of the plants, and also decreased the content of sugars (soluble sugars, reducing sugars, and non-reducing sugars), compared with plants that were grown in soil that was not treated with As. However, the content of proline was increased by increasing the levels of As in the soil, when compared with plants not treated with As (**Figure 5**). The content of various sugars, phenolic acids, and nutrients in the roots and shoots of the plants was determined after the application of *P*. *vermicola* and FeO-NPs. The results suggested that the application of *P*. *vermicola* and FeO-NPs non-significantly increased the sugar content (soluble sugars, reducing sugars, and non-reducing sugars) and proline content in the shoots, and significantly increased the content of nutrients (Ca^2+^, Mg^2+^, Fe^2+^, and P) in the roots and shoots of the plants, compared with plants grown without the treatment of *P*. *vermicola* and FeO-NPs at all levels of As in the soil.

### 3.4 Ameliorative effects of iron oxide nanoparticles and *Providencia vermicola* on organic acids, transmission electron microscopy, and arsenic uptake under arsenic stress

**Figure 8:**
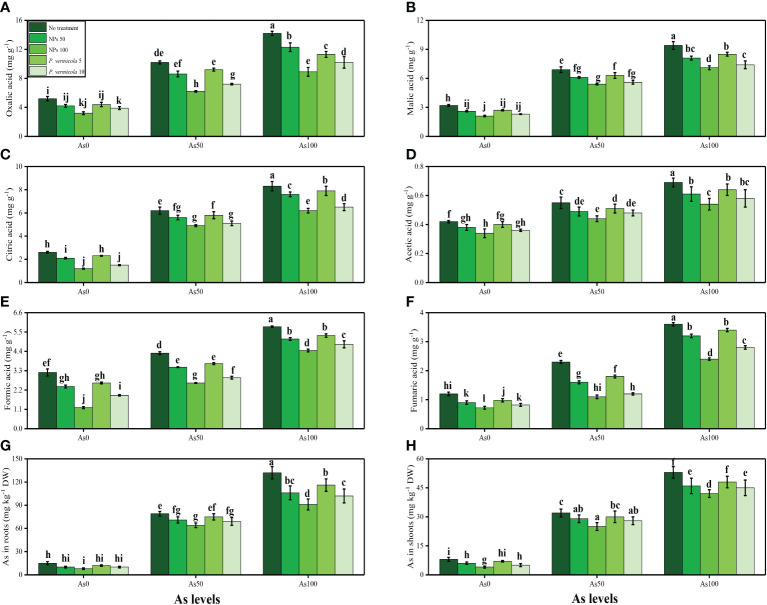
Effect of combined application of various levels of iron oxide nanoparticles (FeO-NPs) (i.e., 50 mg/l^−1^ and 100 mg/l^−1^) and plant growth-promoting bacteria (*Providencia vermicola*) (i.e., 5 ppm and 10 ppm) on oxalic acid contents **(A)**, malic acid contents **(B)**, citric acid contents **(C)**, acetic acid contents **(D)**, formic acid contents **(E)**, fumaric acid contents **(F)**, in the roots and As contents in the roots **(G)**, and As contents in the shoots **(H)** of Ajwain (*Trachyspermum ammi* seedlings) grown under various stress levels of arsenic (i.e., 0 μM, 50 μM, and 100 μM). Values are demonstrated as means of four replicates along with standard deviation (SD; *n* = 4). Two-way ANOVA was performed and means differences were tested by Tukey’s highly significant difference *post-hoc* test (*p*< 0.05). Different lowercase letters on the error bars indicate significant difference between the treatments.

The content of fumaric acid, formic acid, acetic acid, citric acid, malic acid, and oxalic acid in the roots, and As concentration in the roots and shoots, of *T*. *ammi* seedlings grown under toxic levels of As in the soil, with or without the application of *P*. *vermicola* and FeO-NPs, are presented in **Figure 8**. According to the given results, we have noticed that elevated concentrations of As in the soil (i.e., 50 µM and 100 µM) induced a significant (*p*< 0.05) increase in the content of fumaric acid, formic acid, acetic acid, citric acid, malic acid, and oxalic acid in the roots, and also As concentration in the roots and shoots, of *T*. *ammi* seedlings, compared with plants that were grown in the soil treated with 0 µM of As. The results also illustrated that the application of *P*. *vermicola* and FeO-NPs decreased the content of fumaric acid, formic acid, acetic acid, citric acid, malic acid, and oxalic acid in the roots, and also As concentration in the roots and shoots, of *T*. *ammi* seedlings, compared with plants that were grown without the exogenous application of *P*. *vermicola* and FeO-NPs in the soil.

**Figure 9:**
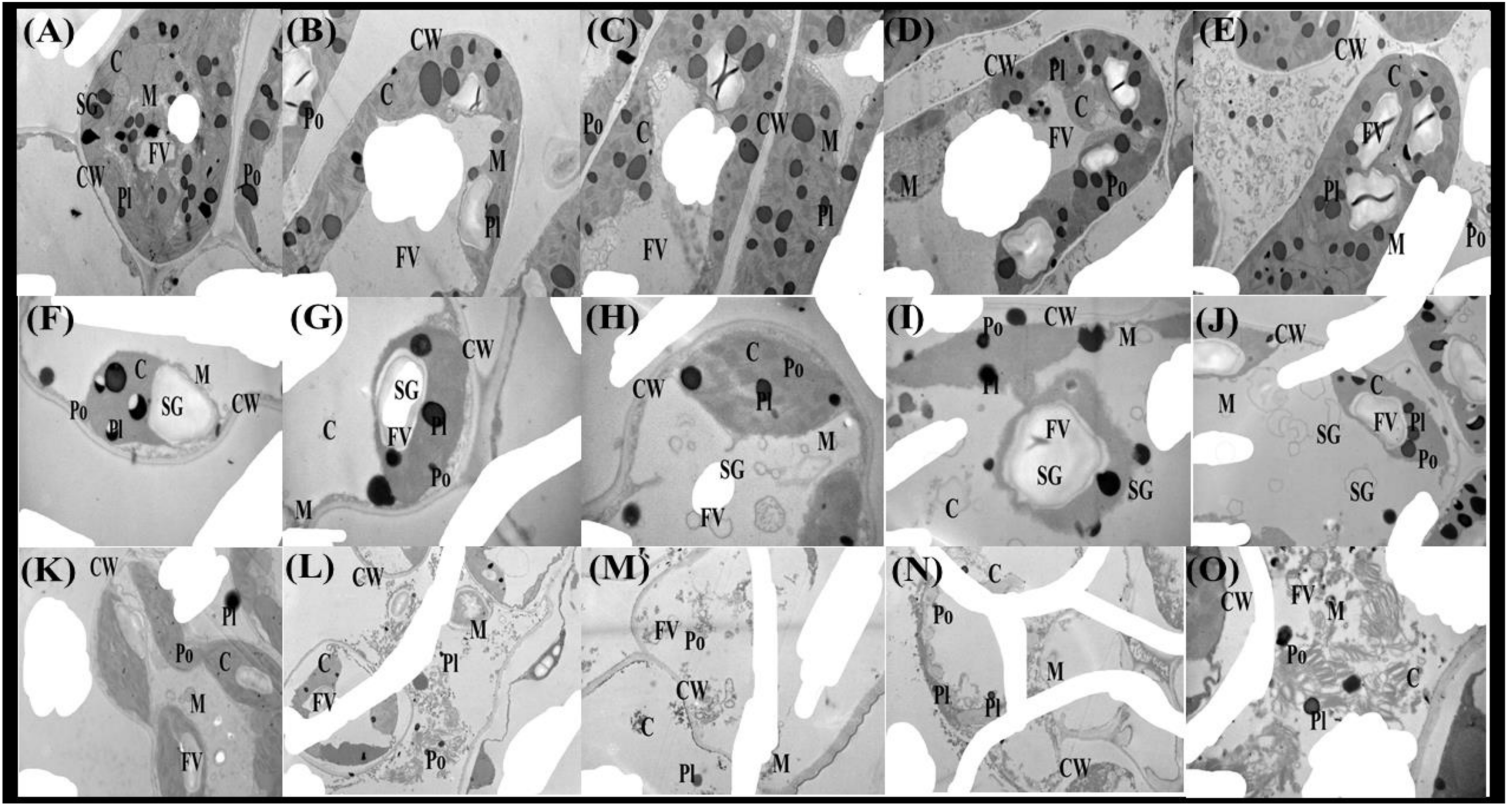
Transmission electron microscopy (TEM) photos of Ajwain (*Trachyspermum ammi* seedlings) grown under various stress levels of As (i.e., 0 μM, 50 μM, and 100 μM). Different lowercase abbreviations in the TEM photos are presented as: C, chloroplast; CW, cell wall; M, mitochondria; FV, food vacuole; SG, starch grain; Po, peroxisome; and Pl, plastoglobuli. Different uppercase abbreviations are used for the various treatments of iron oxide nanoparticles (FeO-NPs) (i.e., 50 mg/l^−1^ and 100 mg/l^−1^) and plant growth-promoting bacteria [*Providencia vermicola* (*P. vermicola*)] (i.e., 5 ppm and 10 ppm) grown under various stress levels of As (i.e., 0 μM, 50 μM, and 100 μM). For example: **(A)** FeO-NPs 0 mg/l^−1^, *P. vermicola* 0 ppm, and As concentration 0 μM; **(B)** FeO-NPs 50 mg/l^−1^, *P. vermicola* 0 ppm, and As concentration 0 μM; **(C)** FeO-NPs 100 mg/l^−1^, *P. vermicola* 0 ppm, and As concentration 0 μM; **(D)** FeO-NPs 0 mg/l^−1^, *P. vermicola* 5 ppm, and As concentration 0 μM; **(E)** FeO-NPs 0 mg/l^−1^, *P. vermicola* 10 ppm, and As concentration 0 μM; **(F)** FeO-NPs 0 mg/l^−1^, *P. vermicola* 0 ppm, and As concentration 50 μM; **(G)** FeO-NPs 50 mg/l^−1^, *P. vermicola* 0 ppm, and As concentration 50 μM; **(H)** FeO-NPs 100 mg/l^−1^, *P. vermicola* 0 ppm, and As concentration 50 μM; **(I)** FeO-NPs 0 mg/l^−1^, *P. vermicola* 5 ppm, and As concentration 50 μM; **(J)** FeO-NPs 0 mg/l^−1^, *P. vermicola* 10 ppm, and As concentration 50 μM; **(K)** FeO-NPs 0 mg/l^−1^, *P. vermicola* 0 ppm, and As concentration 100 μM; **(L)** FeO-NPs 50 mg/l^−1^, *P. vermicola* 0 ppm, and As concentration 100 μM; **(M)** FeO-NPs 100 mg/l^−1^, *P. vermicola* 0 ppm, and As concentration 100 μM; **(N)** FeO-NPs 0 mg/l^−1^, *P. vermicola* 5 ppm, and As concentration 100 μM; and **(O)** FeO-NPs 0 mg/l^−1^, *P. vermicola* 10 ppm, and As concentration 100 μM.

The As toxicity profoundly influenced the double membrane-bound organelles in the cells of *T. ammi* seedlings (**Figure 9**). The ultra-structure of the chlorophyll and other membrane-bound organelles of *T. ammi* seedlings were significantly affected by As toxicity in the soil. The TEM of the leaf cells of *T. ammi* seedlings showed that the progression in the exogenous As levels substantially destructed the ultra-structure of membrane-bound organelles. For instance, organelles, such as chloroplast, starch grain, food vacuole, mitochondria, nucleus, peroxisomes, and plastoglobuli, were clearly visible under 0 μM of As and combined application of FeO-NPs and *P. vermicola*. However, when increasing the As concentration in the soil, the cellular organelles in the plasma of *T. ammi* seedlings were destroyed considerably and ultimately disappeared by elevating levels of As in the soil (**Figure 9**).

## 4 Discussion

Heavy metal contamination of agricultural lands has become an emerging environmental issue worldwide (Saleem et al., 2020b; Saleem et al., 2020f; Afzal et al., 2021; Amna Ali et al., 2021; Farooq et al., 2022). This problem is becoming a serious threat to humans and animals because of the entry of heavy metals in the food web (Salam et al., 2022; Tariq et al., 2022; Ullah et al., 2022). High concentrations of heavy metals significantly reduce plant productivity and crop yields (Javed et al., 2020; Rana et al., 2020; Saleem et al., 2020c; Alsafran et al., 2022a). Arsenite (^+3^) and arsenate (^+5^) are toxic anions of As that are readily absorbed by plants (Shahid et al., 2019) because both arsenate and phosphate share the same transport pathway: As (V) interferes with metabolic processes, and As (III) reacts with sulfhydryl groups (−SH) and tissue proteins, thereby disturbing the enzymatic activities (Irshad et al., 2020; Faizan et al., 2021; Faryal et al., 2022). As has no known essential function in plant growth, and generally causes damaging effects to the plant growth and photosynthetic pigments. Similar results were observed in the present study, which showed that increasing levels of As in the soil (i.e., 50 µM and 100 µM) decreased plant growth and biomass (**Figure 1**), and also decreased the photosynthetic pigments and gas exchange characteristics (**Figure 2**). It is well known that As is non-essential for plants and causes toxicity even at low and moderate concentrations (Bhat et al., 2022; Saleem et al., 2022; Tanveer et al., 2022). As has been described to decrease the chlorophyll biosynthesis in plants, and it has been well-identified that As causes chlorophyll degradation, growth inhibition, nutrient depletion, photosynthesis activity diminution, and membrane disintegration (Liu et al., 2018; Faryal et al., 2022). As also affects the membrane system of chloroplasts, chlorophyll fluorescence, and photosynthetic pigments, thereby reducing photosynthetic activity (Mushtaq et al., 2020; Bhat et al., 2022).

Heavy metals are considered a primary source of injury to the cell membrane, frequently attributing to lipid peroxidation. As a result of metal accumulation, a large number of active free oxygen radicals are formed, which may be the main cause of cell membrane lipid peroxidation, and may also harm the functioning and structure of the cell membrane (Kamran et al., 2019; Rehman et al., 2019; Afridi et al., 2022a; Afridi et al., 2022b). Excessive ROS production causes oxidative stress, as reported for many crops under heavy metals treatment, and is likely to be commenced by molecular oxygen excitation (O_2_) to generate singlet oxygen or by electron transfer to O_2_ and genesis of free radicals (i.e., O^2−^ and OH^−^) (Mfarrej et al., 2021; Bibi et al., 2024). Plant response to oxidative stress also depends on plant species and cultivars, and this ROS production in plants is removed by a variety of antioxidant enzymes, such as SOD, POD, CAT, and APX (Murtaza et al., 2021; Akhtar et al., 2022; Ali et al., 2022a). However, the reduction in antioxidants under severe levels of As in soil might be due to alterations in gene expression and function of various proteins in plant tissues (**Figures 4**, **5**). Plants produce a variety of secondary metabolites, such as proline, flavonoids, and phenolics, that improve tolerance against metal toxicity (Saadullah et al., 2022; Wahab et al., 2022). Previously, an increase in antioxidant activities under elevated levels of As in the soil/medium was found in *Triticum aestivum* (Alamri et al., 2022), brinjal (Alamri et al., 2021), *Brassica napus* (Farooq et al., 2015), and *Myracrodruon urundeuva* (Gomes et al., 2014).

Furthermore, As has been shown to change the nutrient balance and their assimilation, protein metabolism, and oxidative phosphorylation (Mondal et al., 2021; Alsafran et al., 2022b). Excess As decreased the Ca^2+^, Mg^2+^, Fe^2+^, and P in the roots and shoots of the numerous plant species, which may cause ions deficiency in plants (**Figure 7**). Roots exclude especially organic acids, which are regarded as active ligands under the excess concentration of metals in the soil. Acidification of mucilage after uptake of As is likely due to the release of protons when plant roots release more cations than anions to maintain their charge balance (Javed et al., 2020; Sayadi et al., 2022). The exudation of organic acids in the roots of *T. ammi* seedlings (**Figure 8**), accelerating metal transport from roots to the aboveground parts, is possibly due to the formation of metal-chelated ions, as suggested by Javed et al. (Javed et al., 2017) when they cultivated *Zea mays* in Cd-polluted soil. However, using TEM technology, it was revealed that excess As mainly affected many membrane-bound organelles of *T. ammi* seedlings (**Figure 9**).

As compared with their metal oxides NPs, the FeO-NPs are very eco-friendly, non-invasive, low cost, and a safer option to use for various purposes, especially in alleviating the heavy metal-induced oxidative stresses (Mukherjee et al., 2014; Mushtaq et al., 2020). Furthermore, it is reported that the FeO-NPs enhance the nano-fertilizers and nano-materials in the soil that help the plants in the uptake of essential nutrients from the soil (Nair and Chung, 2015; Tanveer et al., 2022). Recently, it was shown that the FeO-NPs could minimize the oxidative stresses in Cd-stressed *Triticum aestivum* (Rizwan et al., 2019) and As-stressed *Oryza sativa* (Rai et al., 2022). On exposure to different levels of FeO-NPs, the plants experienced lower metal uptake and, thus, resulted in lower cellular damage (Hussain et al., 2019; Mushtaq et al., 2020). A study on various parts of the plants demonstrated that the exogenous application of nano-iron (II, III) oxide (200 mg/l^−1^) alleviated the Pb-, Zn-, Cd-, and Cu-induced toxic effects by stimulating the various antioxidant activities that resulted in reduced metal uptake (Konate et al., 2017). In a hydroponic study, the exogenous application of nano-iron (II) oxide (50 ppm) to mung bean (*Vigna radiate* L.) induced the increment in growth and biomass, indicating significant roles of the nano-particles in improving plant growth under normal and various stress conditions (Dhoke et al., 2013). Our outcomes are corroborated with the aforementioned studies, as exogenous application of FeO-NPs improved the plant growth (**Figure 1**), photosynthetic characteristics (enzymatic and non-enzymatic antioxidants), gene expression, sugars, and essential nutrients, and maintained the ultra-structure of the membrane-bound organelles by considerably scavenging the ROS levels, organic acid in the roots, and As accumulation.

**Figure 10:**
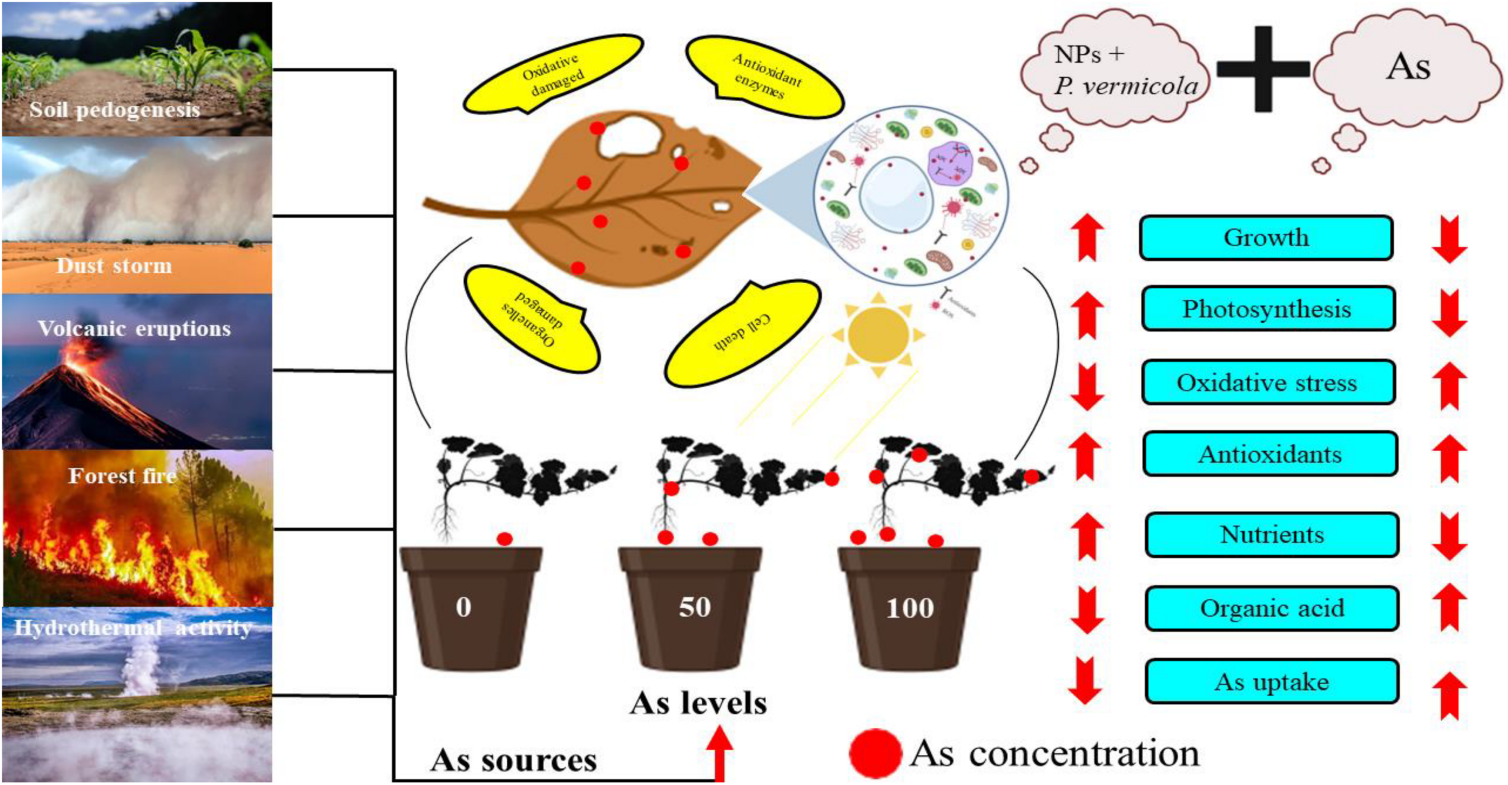
Schematic presentation of the findings from this study under the application of *Providencia vermicola* (*P. vermicola*) and iron oxide nanoparticles (FeO-NPs) in arsenic (As)-stressed Ajwain [*Trachyspermum ammi* (*T*. *ammi*)] seedlings grown under different levels of As stress (i.e., 50 μM and 100 μM) in sandy loam soil. The figure shows As sources in the natural soil and its toxic effects on the plants. The figure also shows that As toxicity can be overcome by the application of *P. vermicola* and FeO-NPs, which decreased oxidative stress in membrane-bound organelles by decreasing As content in various parts of the plants. Overall, this scheme presents the complete description of this experiment and the important findings that we have evaluated from the application of *P. vermicola* and FeO-NPs in As-stressed Ajwain (*T*. *ammi*) seedlings.

Like NPs, PGPB have been identified to cope with various heavy metal-induced toxicity by improving plant growth and development in metal-contaminated soil (Kaushal and Wani, 2016). Generally, it was noticed that the PGPB are involved in the chlorophyll formation, protein biosynthesis, activating the antioxidant potential, up-regulating the production of osmolytes, root formation, extend leaf area, and greater nutrients uptake, and, therefore, increases crop productivity (Kasim et al., 2013; Danish et al., 2019; Mondal et al., 2021). Various PGPB, such as *Pseudomonas*, *Bacillus*, *Streptomyces* and *Agrobacterium*, are commercialized and easily available in the market, and produce antibiotics that enable plants to resist any pathogen attack (Hussain et al., 2015). Earlier, the same strains of *P*. *vermicola* were used by Islam et al. (Islam et al., 2016) in lentil plants. Islam et al. noticed that the *P*. *vermicola* exhibited multiple growth-promoting characteristics by improving photosynthetic machinery and increasing plant yield, which helped the lentil plants to mitigate the toxic effects of Cu-induced oxidative stress. The same strains of *P*. *vermicola* coupled with NPs were also used by Tanveer et al. (Tanveer et al., 2022). Tanveer et al. found that the application of (10-ppm) *P*. *vermicola* considerably enhanced the proline content, relative water content, sugar protein, and indole acetic acid in As-stressed *Luffa acutangula*. The authors further noticed that the *P*. *vermicola* substantially decreased the relative EL and As concentration in *Luffa acutangula* and, thus, improved the overall growth of the plants. *P*. *vermicola* also has the ability to produce siderophores and the ability to uptake essential ions, such as P, K and, Ca, to produce efficient bioinoculants, which were revealed under the biochemical analysis of *P*. *vermicola* (Hussain et al., 2015). Soil inoculation with *P*. *vermicola* enhanced the activities of enzymatic and non-enzymatic antioxidant compounds by producing defensive enzymes, such as glutathione reductase, which captures the extra ROS produced under metal toxicity (Zuo et al., 2018). Like the aforementioned reports, our study revealed that the use of PGPB (*P*. *vermicola*) coupled with FeO-NPs remarkably improved the nutrient uptake and efficacy of the photosynthetic machinery that assisted the *T. ammi* seedlings in maintaining their cellular structure under As-induced oxidative stress, thus, potentially activating the antioxidant potential and their respective transcript levels, which ultimately led to the improved plant growth. Similar results were obtained by Ahemad, Hofmann et al., and Tanveer et al. (Ahemad, 2019; Hofmann et al., 2020; Tanveer et al., 2022) under different environmental conditions, signifying the fact that the effects of various PGPB and NPs mainly depend on the plant species, soil composition, method of application, and amount and length of the treatments. These results further open the doors for future scientists to focus on the aforementioned factors to gain deeper insights into the role of PGPB and NPs in ameliorating the oxidative damage caused by heavy metal-induced toxicity. The schematic presentation of mechanistic role of *P. vermicola* and FeO-NPs alleviating the As toxicity in *T*. *ammi* seedlings is presented in **Figure 10**.

## 5 Conclusion

On the basis of these findings, it can be concluded that the negative impact of As toxicity can be overcome by the external application of *P. vermicola* and FeO-NPs. Our results depict that As toxicity induced severe metal toxicity in *T*. *ammi* seedlings by increasing the generation of ROS in the form of oxidative stress, and also increased the concentration of As in the roots and shoots of the plants. Furthermore, As toxicity also increased organic acids exudation and imbalance the nutritional status of the plants and destroy the ultra-structure of the plants, which ultimately decrease plant growth and yield and photosynthetic efficiency. Hence, As toxicity was eliminated by the application of *P. vermicola* and FeO-NPs, which also decreased the As concentration in the plant tissues, degenerated ROS, induced ultra-structure alterations and organic acids exudation, and increased the activities of antioxidants and essential nutrients in the plants. Therefore, long-term field studies should be executed to draw parallels among plants/crops root exudations, metal stress, Fe fertigation regimes, nutrients mobility patterns, and plant growth to gain insights into underlying mechanisms.

## Data availability statement

The original contributions presented in the study are included in the article/**Supplementary Material**. Further inquiries can be directed to the corresponding author.

## Author contributions

Formal analysis, YS; Methodology, JM and BL; Project administration, CF; Software, CF; Supervision, YZ and CF; Writing – original draft, YS, LM, and JM; Writing – review and editing, YS, LM, JM, YZ, and CF. All authors have read and agreed to the published version of the manuscript.
