# Supplementary material for: Combined application of plant growth-promoting bacteria and iron oxide nanoparticles ameliorates the toxic effects of arsenic in Ajwain (Trachyspermum ammi L.)
Source: Front Plant Sci. 2022 Dec 28;13:1098755. doi: 10.3389/fpls.2022.1098755 (PMC9832315; doi:10.3389/fpls.2022.1098755)
Supplement: Supplementary file 1 [file Table_1.docx]

**Table S1** Physical and nutritional properties of the soil used in this experiment

| ­­­­­­­**Physical properties** |  |
| --- | --- |
| Organic matter | >70% of total solids |
| Density | 350 Kg/m^3^ |
| pH | 7.6 |
| Electrical conductivity | 20mS/m |
| Organic nitrogen | 1400 mg/L |
| **Nutrients** | **g/m^3^** |
| Nitrogen (NO_3_-N + NH_4_-N) | 150 |
| Phosphorus (P) | 75 |
| Potassium (K) | 160 |
| Magnesium (Mg) | 250 |
| Calcium (Ca) | 1600 |
| Sulphur (S) | 85 |
| Copper (Cu) | 2.5 |
| Zinc (Zn) | 1.8 |
| Molybdenum (Mo) | 2.7 |
| Iron (Fe) | 5.6 |

**Table 2S** Gene-specific primers sequences used in the present study

| Gene | Primer Sequence (5′-3′) | Gene Accession Number | Reference |
| --- | --- | --- | --- |
| Fe-SOD | F: ATCTTAGTTATGGTTCTCTTTGT  R: ATGGTGTAGAGCCTTTTCATAT | M64267 | [62] |
| POD | F: TTGAAATAAAC CAAAGGAGTAGT  R: AATAATTATTTGAATCTCTTTAAGG | AF145349 | [62] |
| CAT | F: AGCATCTCACCTGAACTTGAA  R: AGGTGAGAGGTTTGTGGCC | AF035252 | [62] |
| APX | F: CGTGACGATGATTGGGAAGT  R: TGATAGTGATCTTTCGGACCT | NM_001354113 | [62] |
